# A Novel Strategy to Produce a Soluble and Bioactive Wheat Bran Ingredient Rich in Ferulic Acid

**DOI:** 10.3390/antiox10060969

**Published:** 2021-06-16

**Authors:** Ana Belén Martín-Diana, Irene Tomé-Sánchez, María Jesús García-Casas, Cristina Martínez-Villaluenga, Juana Frías, Daniel Rico

**Affiliations:** 1 Agricultural Technological Institute of Castile and Leon (ITACyL), Government of Castile and Leon, Ctra. de Burgos Km. 119, Finca Zamadueñas, 47071 Valladolid, Spain; garcasmj@itacyl.es (M.J.G.-C.); ricbarda@itacyl.es (D.R.); 2 Institute of Food Science, Technology and Nutrition (ICTAN-CSIC), José Antonio Novais 10, 28040 Madrid, Spain; i.tome@ictan.csic.es (I.T.-S.); c.m.villaluenga@csic.es (C.M.-V.); frias@ictan.csic.es (J.F.)

**Keywords:** wheat bran, hydrothermal treatment, high hydrostatic pressure, spray-drying, microencapsulation, total antioxidant activity, anti-inflammatory activity

## Abstract

Wheat bran (WB) is a byproduct from the milling industry that contains bioactive compounds beneficial to human health. The aim of this work was on the one hand, increasing extractability of antioxidant and anti-inflammatory compounds (specifically ferulic acid, FA), through enzymatic hydrolysis combined with hydrothermal treatment (HT) and high hydrostatic pressure (HHP). On the other hand, enhancing the stability of final ingredient applying spray-drying (SPD) and microencapsulation (MEC). The use of HT increased FA, total phenolics (TP), and antioxidant capacity (AC) in WB hydrolysates, regardless the HT duration. However, the HT tested (30 min, HT30) produced a loss in anti-inflammatory activity (AIA). The combination of HT (15 min, HT15) with HHP increased AIA of the WB. SPD enhanced the TP yield in WB with no significant effect of inlet temperature (up to 140 °C) on phenolic profile mainly composed of *trans-*FA and smaller amounts of *cis*-FA and apigenin diglucosides. SPD caused a temperature-dependent increase in AC (160 °C > 140 °C > 130 °C). SPD inlet temperatures affected total solids yield (from 22 to 36%), with the highest values at 140 °C. The use of HHP in combination with HT resulted in >2-fold increase in total solids yield.

## 1. Introduction

The growing interest in a healthy and balanced diet by consumers has increased the demand for naturally functional foods and nutraceuticals with potential health attributes. The global functional food market size was valued at USD 382.51 billion in 2019 and it is expected to increase at a CAGR of 8.3% over the next years [1]. Nutraceuticals occupy the niche that exists between food and medicine, being currently categorized as dietary supplements by the European Food Safety Authority (EFSA), who defines them as “concentrated sources of nutrients or other substances with a nutritional or physiological effect that can be commercialized in “dose form” [2]. When added to food as ingredients, they are affected by Regulation (EC) n. 1924/2006, regarding nutrition and health claims on food [3]. Bioactive ingredients can be obtained from different sources, being plant materials the most popular products for the extraction of bioactive compounds due mainly to their high content in polyphenols.

The production of an ingredient implies a significant processing cost, for this reason, selecting an abundant and affordable natural source is quite important. In this sense, the cereal industry produces an important volume of byproducts during grain milling, where bran represents about 15% of the grain weight, therefore, bran production is still an issue of environmental concern [4]. Although applications for wheat bran (WB) exist in the food industry, traditionally it has been mostly used as a low-value ingredient for animal feed purposes, meanwhile other brans such as oat bran has gained popularity in human diet.

WB is not only an excellent source of nutrients (13–18% protein, 3.5% fat, and 56% carbohydrates of which 70–90% is dietary fiber, DF) but also of bioactive components (tocopherols, phenolic compounds, carotenoids, etc.) [5,6,7,8,9,10] with recognized health benefits. Moreover, different authors have reported the important antioxidant activity of WB [7,9,11,12] and many studies have associated these properties to health benefits such as protection against low-density lipoprotein (LDL) oxidation system [13]. Additionally, it has been suggested that the antioxidant properties in WB may modulate cellular oxidative status and prevent biological oxidative damage, and that this consequently plays a role in reducing the risk of chronic diseases such as cardiovascular diseases (CVDs) and cancer [14]. All this existing evidence has recently promoted the interest to explore new applications of WB in the development of functional ingredients. A recent comparative study from our research group pointed out the nutritional advantages of WB with respect to oat bran that can be summarized in higher amounts of total DF and phenolic compounds, with greater antioxidant capacity and lower glycemic index, supporting the use of WB in the formulation of functional foods [11].

Up to date, two health claims regarding WB DF consumption has been approved by the EFSA [15]. WB polysaccharides can increase fecal bulk and modulate the intestinal transit time through the water-binding capacity of the large polymer network. Additionally, colonic microbial fermentation of soluble and insoluble (in a lesser extent) DF fractions of WB is associated to an increased production of short chain fatty acids and, consequently, beneficial health effects have been recognized [16]. Furthermore, arabinoxylans (AX), the main polysaccharide of WB DF (18.8–21.4% of WB dry weight), have demonstrated to exert immunomodulatory effects by activating immune system cells [17].

WB is rich in phenolic acids (cinnamic and benzoic acids) and flavonoids that mainly appear as glycosides, linked to sugar moieties [11]. Phenolic acids are the most representative phenolic group in WB, being ferulic acid (FA) the most abundant compound followed by minor amounts of sinapic and *p*-coumaric acids. Up to the 95% of FA in WB is covalently linked to AX and lignin [8,9]. FA has demonstrated antioxidant and anti-inflammatory effects in vitro and in vivo, properties well exploitable in the food, health, and cosmetic sectors [18]. 

Despite the potential health benefits associated with WB consumption, it can be assumed that the rigid structure and strong association of WB components (cellulose, AX, lignans, and phenolic compounds) limit their capability to exert their physiological action. Therefore, technological strategies aimed to solubilize phenolic acids from the cell wall components need to be addressed for the functionalization of WB by improving the bioaccessibility of bioactive components. Possible strategies include mechanical treatments, for particle size reduction, heat-pressure treatments and bioprocessing (enzymatic hydrolysis and fermentation) for DF solubilization and phenolic compounds release [8,19,20,21,22]. The use of enzymatic treatments was performed by our research group to improve the extraction and solubilization of bioactive compounds from WB [23]. In particular, sequential hydrothermal (HT) and enzymatic treatments of WB with Ultraflo XL at optimal temperature, pH and time maximized free FA content and its capacity to scavenge oxygen radicals, chelate transition metals, and inhibit secretion of cytokines in a cellular model of inflammation [9].

Recent studies have reported the efficiency of thermal treatments such as hydrothermal and or extrusion to solubilize DF components and phenolic compounds entrapped in the DF fraction [7,20]. Limitations of the use of thermal treatments linked to significant loss of thermolabile phenolic compounds in WB open new opportunities to explore the potential use of non-thermal processing such as high hydrostatic pressure (HHP) as an alternative method for the functionalization of WB. HHP can solubilize DF components in plant-based foods including WB [21] although it remains to be studied the effect of this non-thermal treatment on the phenolic fraction of WB and whether these effects have an impact in the antioxidant and immunomodulatory potential of final products. Combination techniques like HT and HHP could also be promising approximations in the functionalization of WB to reduce processing time and making the process more energy-efficient [22]. Additionally, the use of organic acids have gained interest in the food industry for their recognized antimicrobial properties and their safe designation [23,24,25,26].

WB technological transformation in value-added products must include stabilization methods to guarantee their stability. Several studies have pointed out the instability of phenolic compounds to temperature, light, humidity, and oxygen [27,28]. Spray-drying (SPD) could be a suitable process to stabilize WB bioprocessing downstream products into a solid powder. This technology is simple, relatively inexpensive, rapid, and, thus, widely used in industry. However, some disadvantages have been also reported including the loss of a significant amount of product and the possibility of degradation of sensitive products at high drying temperatures. The extent of these changes depends on the intensity of the SPD process [29,30], which can be optimized by adjusting operating conditions. Additionally, SPD can be used as microencapsulation (MEC) technique with the incorporation of encapsulating agents (e.g., plant proteins isolates) in the process, affecting positively to the stability of microparticles, the process efficiency, and the degree of protection of bioactive compounds [31]. Therefore, the aim of the present work was to gain insight into the effect of a multistep process combining HT and HHP, and a further step of enzymatic hydrolysis using different organic acids and SPD and MEC for stabilization of WB hydrolysates rich on FA with antioxidant and immunomodulatory properties. 

## 2. Materials and Methods

### 2.1. Chemicals

2,2′-Azinobis 3-ethylbenzothiazoline-6-sulfonic acid (ABTS), 2,2′-diazobis-(2-aminodinopropane)-dihydrochloride (AAPH), fluorescein, 2,2-diphenyl-1-picrylhydrazyl (DPPH), Folin–Ciocalteu (FC) reagent, gallic acid (GA), and 6-hydroxy-2,5,7,8-tetramethyl-2-carboxylic acid (Trolox) were obtained from Sigma-Aldrich, Co. (St. Louis, MO, USA). All the solvents were HPLC grade (Sigma Aldrich Co., Madrid, Spain, and Merck KGaA, Darmstadt, Germany). Chlorogenic acid, 2-coumaric acid, 2,4-dihydroxybenzoic acid, 4-hydroxybenzoic acid, vanillic acid, and vanillin standards were purchased from Extrasynthese (Lyon, Genay Cedex, France) and caffeic acid, ferulic acid, and vitexin standards were obtained from Sigma-Aldrich, Co. (Madrid, Spain). 

### 2.2. Materials

Wheat (*Triticum aestivum* L. var. Craklin) bran of <800 μm particle size was kindly supplied by Emilio Esteban, S.A. (Valladolid, Spain), and stored in polyethylene bags under vacuum until use. Food grade enzyme Ultraflo XL and Pisane C_9_ protein were kindly provided by Novozymes (Bagsværd, Copenhagen, Denmark) and Innovafood (Barcelona, Spain), respectively.

### 2.3. Chemical Composition of WB

The moisture content was assessed by drying 5 g of powdered sample at 105 °C for 3 h. Total protein content was determined by the Dumas method, 990.03 [32] in an elemental analyzer (LECO Corp., St. Joseph, MI, USA). A conversion factor of 6.25 was used to convert nitrogen into protein values. Total fat content was determined by petroleum ether extraction (40–60 °C) for 4 h using a Soxtec extracting unit (AOAC 2005, method 2003.05) [32]. 

Fatty acid profile was determined for WB. The Blight and Dyer method [33] was used for lipid extraction. Lipid-containing chloroform phase was separated and evaporated. The remaining phase was dissolved in 1 mL of hexane, and a methylated procedure was carried out by adding 100 μL of 0.5 M methanolic KOH and leaving the reaction for 10 min at room temperature (RT). The upper layer was transferred to a 2 mL vial. Analysis of fatty acid methyl esters (FAME) was carried out on a gas chromatograph Agilent 7890A (Agilent Technologies, Santa Clara, CA, USA) and a flame ionization detector. For the analysis, the method was run from 50 to 200 °C for the first 7 min at a rate of 3 °C min^−1^ and held for 26 min. Injector and detector temperatures were 250 °C and 280 °C, respectively. After, 1 μL of the hexane extract was injected in split mode (ratio 25:1), and FAMEs were identified by comparison of retention times with those of 37 FAMEs standard mix (Supelco, Sigma-Aldrich, St. Louis, MO, USA). Fatty acid profile was expressed in percentage respect to total fatty acids.

Ash content was determined by sample incineration in a muffle furnace at 550 °C for 5 h (AOAC 2005, method 923.03) [32]. Total dietary fiber, β-glucan, total starch, and phytic acid/total phosphorus content were determined using the K-RINTDF, K-BGLU, K-TSTA-100A, and K-PHYT assay kits (Megazyme, Wicklow, Ireland), respectively. All results were corrected for moisture content and expressed as g 100 g^−1^ of dry matter (d.m.). All analyses were carried out in duplicate.

### 2.4. WB Processing Route

**Figure 1:**
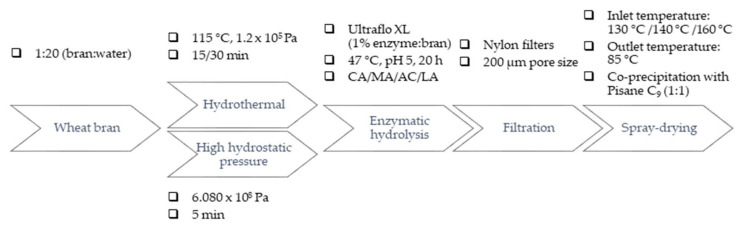
Processing route and operating conditions for the release of phenolic compounds and functionalization of WB. Abbreviations: citric acid (CA); malic acid (MA); acetic acid (AC); lactic acid (LA).

An overview of the processing route and process parameters applied to native WB is shown in Figure 1. Initially, 200 g of WB were suspended in 2 L water (1:20 *w*/*v*). In a first step, the effect of HT time was evaluated. WB suspension was firstly thermally treated using an Ilpra Plus 100 autoclave equipment (Ilpra Systems, Barcelona, Spain) at 115 °C, 1.2 × 10^5^ Pa following the condition studied in previous work for the authors [7] at two different times (15 and 30 min). In a second step, the effect of HHP was evaluated, the WB mixture was pressurized (6.08 × 10^8^ Pa, 5 min) before or after the HT, using a HHP unit (Wave 6000/135, NC Hyperbaric, Burgos, Spain) with a vessel of 135 L and 200 mm diameter. The temperature of the water inside the chamber was monitored during the processing. Third step, different organic acids (citric (CA), malic (MA), acetic (AC), and lactic acid (LA)) were used to adjust pH value to 5 before enzyme incorporation. Immediately after, Ultraflo XL was added at 1% (enzyme to WB dry weight ratio, *w*:*w*) and enzymatic hydrolysis was performed at 47 °C, pH 5 for 20 h using a temperature-controlled water-bath with magnetic stirring set at 1000 rpm (Unitronic Vaivén C, Selecta S. A., Spain). The processing conditions were selected based on our previous optimization study aimed to maximize the antioxidant and anti-inflammatory properties of WB through solubilization of DF and release of FA [9]. At the end of incubation period, enzyme was inactivated submerging WB hydrolysates in a water bath at 95 °C for 10 min. Insoluble residues were removed by filtration using a nylon filter (200 μm-mesh). Finally, WB soluble fraction (filtrate) was stored at 4 °C until further analysis.

Soluble fraction of WB hydrolysate was stabilized by SPD. This process was carried out at pilot scale in the facilities of the Institute of Bioeconomy of the University of Valladolid (Spain). The liquid product was introduced into a MM basic rotary spray dryer (GEA Mobile Minor™_,_ Düsseldorf, Germany) and came into contact with a stream of pressurized (6.08 × 10^8^ Pa) and heated air. The effect of inlet air temperature was studied at 130, 140 and 160 °C using an air flow rate at 114 m^3^ h^−1^. The outlet temperature was fixed at 85 °C using a flow rate of 0.78 L h^−1^, according to Salgado et al. [34]. The equipment was run for 15 min with distilled water before each experiment in order to reach a stable outlet temperature. The WB mixture was constantly stirred and pumped into the equipment using a peristaltic pump (Watson Marlow 520S, Wilmington, MA, USA). The powder was collected at the exit in a cyclone, discarding any microparticles deposited on the dryer chamber and transferred into sealed glass containers and stored until further used.

For microencapsulation (MEC), WB hydrolysate was mixed with Pisane C_9_ (pea protein isolate with 86% protein of high solubility) used as encapsulating agent in a ratio 1:1 of pea protein: WB hydrolysate dry weight [35]. For encapsulation, inlet air temperature was selected at 130 °C, and the outlet air temperature was maintained at 85 °C using a WB flow of 0.78 L h^−1^. The microencapsulated WB was recovered and transferred into sealed glass containers and stored until further used.

### 2.5. Water Activity

Water activity (*a_w_*) of WB obtained after SPD and MEC experiments was measured with a fast water activity meter (Aqualab 4TE, Decagon Devices Inc., Pullman, WA, USA). A_w_ was evaluated as the partial pressure of water vapor in a substance divided by the standard state partial pressure of water vapor. Values were expressed from 0 to 1.

### 2.6. Quantification of Total Phenolics Compounds (TP)

Free phenolic compounds were extracted using ethanol/water (80:20, *v*:*v*) as described in Bautista-Expósito et al. [9]. Bound phenolics were extracted by sequential alkaline and acid hydrolysis following the procedure described by Dinelli et al. [36]. TP content was measured in soluble and insoluble fractions using the Folin–Ciocalteu phenol reagent, according to Slinkard and Singleton [37], with modifications [38]. The absorbance was measured at 765 nm using a microplate reader (Fluostar Omega, BMG, Ortenberg, Germany). Gallic acid was used as the standard (500–100 µM). Results were expressed as mg of gallic acid equivalents (GAE) 100 g^−1^ d.m. All the analyses were carried out in duplicate.

### 2.7. High-Performance Liquid Chromatography Electrospray Ionization-Quadrupole-Time-of -Flight Mass Spectrometry (HPLC-ESI-QTOF/MS) Analysis

Phenolic compounds characterization was performed using a high-performance liquid chromatograph (HPLC) (model 1200 series, Agilent Technologies, Santa Clara, CA, USA) equipped with a diode array detector (model G1315B, Agilent Technologies, Santa Clara, CA, USA) coupled with an accurate-mass quadrupole-time of flight mass spectrometer (Agilent G6530A, Agilent Technologies, Santa Clara, CA, USA) with an electrospray-ionization source (HPLC-ESI-QTOF-MS). The phenolic fractions were injected (20 µL) and separated in an Agilent ZORBAX Eclipse XDB-C_18_ analytical column (4.6 mm × 150 mm × 5 µm) (Agilent Technologies, Santa Clara, CA, USA) at 40 °C followed by detection by Diode Array Detection (DAD) at 280 nm and 360 nm. Two mobile phases were prepared including water/formic acid (99.9:0.1, *v*:*v*; eluent A), and mobile phase B consisted of acetonitrile/formic acid (99.9:0.1, *v*/*v*; eluent B). The gradient of elution was kept for 40 min at a flow rate of 1 mL min^−1^ as follows: in 20 min, from 5% to 15% B; in 10 min, from 15% to 30% B; in 5 min, from 30% to 50% B; in 2 min, from 50% to 5% B. At the end of the elution gradient, the mobile phase composition was back to initial conditions and the column was equilibrated for 3 min before next injection. For the identification and quantification of components, mass spectrometry (MS) and tandem mass spectrometry (MS/MS) runs were performed in the negative ion mode. Mass spectra in the *m*/*z* range 100–1200 were obtained. The MS conditions were set as follows: nitrogen gas temperature was fixed at 325 °C and a flow rate of 10 L min^−1^, sheath gas temperature was set at 300 °C and a flow rate of 11 L min^−1^ and nebulizer gas pressure was set at 45 psi. The capillary, nozzle, and fragmentation voltages were set as 4000 V, 0 V, and 125 V, respectively. For targeted MS/MS experiments fixed collision energy used was 20 V. Data acquisition and processing was carried out using MassHunter Data Acquisition (version B.05.00) and Qualitative Analysis (version B.07.00) Workstations (Agilent Technologies, Waldbroon, Germany). Quantification of phenolic compounds was carried out using calibration curves of authentic standards (caffeic acid, chlorogenic acid, 2-coumaric acid, 2,4-dihydroxybenzoic acid, ferulic acid, gallic acid, 4-hydroxybenzoic acid, vanillic acid, vanillin, and vitexin) at a concentration range between 0 and 25 µg mL^−1^) showing good linearity (*R*^2^ > 0.99). Data were expressed as mean and standard deviation of two independent replicates in mg 100 g^−1^ d.w. (dry weight). 

### 2.8. Total Antioxidant Capacity (TAC)

TAC was measured using DPPH radical scavenging activity; oxygen radical absorbance capacity (ORAC); ABTS radical cation scavenging activity, and ferric reducing antioxidant power (FRAP). Relative antioxidant capacity index (RACI) was also calculated. All the analyses were carried out in duplicate.

#### 2.8.1. DPPH Radical Scavenging Activity

Antioxidant activity against the DPPH radical was estimated according to the procedure described by Brand-Williams et al. [39] with some modifications. An amount of 100 µL of extracts was mixed with 400 µL of MilliQ water and 500 µL of DPPH working solution (120 µM using methanol as solvent) in a 96-well microplate. Absorbance at 515 nm was recorded for 30 min in a microplate reader (Fluostar Omega, BMG Ortenberg, Germany). Trolox was used as the standard (7.5–210 µM). The results were expressed as µmol Trolox equivalents (TE) 100 g^−1^ d.m. 

#### 2.8.2. Oxygen Radical Absorbance Capacity (ORAC) 

The procedure was based on a previously reported method with slight modifications [40]. The standard curve of Trolox (7.5–180 µM) and all the fractions were diluted in phosphate buffer (75 mM, pH 7.4). A volume of 150 μL fluorescein was placed in 96-well black polystyrene plates, and 25 μL of Trolox standard, sample, or phosphate buffer as blank were added, all in duplicate. Samples, standards, and blanks were incubated with fluorescein at 37 °C for 3 min before AAPH solution was added to initiate the oxidation reaction. Fluorescence was monitored over 100 min using a microplate reader (Fluostar Omega, BMG Ortenberg, Germany) at excitation and emission wavelengths of 485 and 528 nm, respectively. Results were obtained by external calibration, plotting the areas under the fluorescein decay curves as function of Trolox concentration. Data were expressed as µmol TE 100 g^−1^ d.m. 

#### 2.8.3. ABTS^•+^ Radical Cation Scavenging Activity

The antioxidant capacity against diammonium salt of ABTS^•+^ radical was evaluated according to the method first described by Re et al. [31], and lately [41], modified by Martin-Diana et al. [38]. First, 100 µL of diluted fractions were mixed with 1000 µL of ABTS^•+^ working solution in an Eppendorf tube. The decay in absorbance at 734 nm was recorded over 60 min with a microplate reader. Trolox was used as the standard (7.5–210 µM). The absorbance was measured at 734 nm with a microplate reader (Fluostar Omega, BMG Ortenberg, Germany). Results were expressed as µmol TE 100 g^−1^ d.m. 

#### 2.8.4. Ferric Reducing Antioxidant Power (FRAP) 

FRAP assay was performed following the protocol reported by Pereira et al. [42]. Absorbance at 700 nm was recorded in a microplate reader (Fluostar Omega, BMG Ortenberg, Germany). FeSO_4_·7H_2_O was used as a standard (2.4–3.8 mM). The results were expressed as µmol Fe^2+^ equivalents 100 g^−1^ d.m. 

#### 2.8.5. Relative Antioxidant Capacity (RACI)

RACI was used as an integral concept, which allows the comparison of antioxidant capacity derived from different chemical methods [43]. RACI values were determined through the following equation: (x − µ)/σ, where x is the antioxidant value, µ is the average value of the results of the corresponding method (ABTS, ORAC, DPPH, and FRAP), and σ is the standard deviation.

### 2.9. Determination of Anti-Inflammatory Activity (AIA)

Murine macrophage cell line RAW 264.7, provided by ATCC (ATCC^®^ TIB-71^TM^, Rockville, MD, USA), was cultured in Dulbecco’s modified Eagle’s medium (DMEM) (containing 4.5 g L^−1^ with L-glutamine) acquired from Lonza Group (Lonza, Madrid, Spain), and supplemented with 100 U mL^−1^ penicillin, 100 mg L^−1^ streptomycin, and 10% fetal bovine serum (FBS) purchased from Hyaclone (GE Healthcare, Logan, UT, USA). Cells were maintained in an incubator with a 5% CO_2_ humidified atmosphere at 37 °C. Cells were seeded in 96-well plates at a density of 5 × 10^4^ cells/well and cultured overnight at 37 °C with 5% CO_2_. Cells were exposed to soluble fraction of WB hydrolysates at two different doses (0.05 and 0.5 mg mL^−1^ of complete DMEM containing 0.1% FBS). Treated cells were incubated at 37 °C with 5% CO_2_ for 23 h. After incubation, spent medium was replaced by 100 µL of serum free DMEM followed by addition of 20 µL of the Cell Titer 96 Aqueous One Solution Proliferation Assay kit (Promega Biotech Ibérica, Madrid, Spain). After 45 min of incubation at 37 °C with 5% CO_2_, the 96-well plates were read at 490 nm on a Synergy HT microplate reader (Biotek Instruments, Winooski, VT, USA). The cell viability was calculated as the percentage of control (untreated) cells. Data represent the mean and standard deviation of five biological replicates (Figure S3).

The murine macrophage cell line RAW 264.7 was seeded into 96-well plates a density of 5 × 10^4^ cells/well and incubated in complete DMEM at 37 °C and 5% CO_2_ for 48 h. Cells were exposed at two doses of WB hydrolysates (0.05 and 0.5 mg mL^−1^ of complete DMEM supplemented with 0.1% of FBS) for 1 h after which an inflammatory response was induced by addition of 40 ng mL^−1^ of lipopolysaccharide (LPS) from *Escherichia coli* O55:B5 (Sigma-Aldrich, St. Louis, MO, USA). Plates were incubated at 37 °C and 5% CO_2_ for 20 h. Supernatant were collected, stored at −80 °C for quantification of interleukin (IL)-6 and tumor necrosis factor-alpha (TNF-α) concentration. Cytokine quantification was carried out using murine IL-6 and TNF-α enzyme-linked immunosorbent assay (ELISA) kits (Diaclone, Besacon Cedex, France). The absorbance was measured in a Synergy HT microplate reader (Biotek Instruments, Winooski, VT, USA) at 450 nm. Results were expressed as concentration of IL-6 and TNF-α in pg mL^−1^. Data represent the mean and standard deviation of five biological replicates. 

### 2.10. Statistical Analysis

Data were expressed as the mean ± standard deviation. Analysis of variance (ANOVA) and post hoc Duncan’s test was used to identify differences between mean values. Pearson correlation coefficients and principal component analysis (PCA) were performed on centered and standardized data to elucidate the relationships among variables of the phenolic profile and antioxidant capacity of samples. All statistical analyses were performed using Statgraphics Centurion XVI^®^ (StatPoint Technologies, Inc., Warrenton, VA, USA).

## 3. Results and Discussion

### 3.1. Chemical Composition of WB

As expected, WB showed an interesting nutritional value due to its high content in DF and protein (Table 1). The main component of WB was DF that accounted for 46.6 g 100 g^−1^, values within the range reported in the literature [7,44,45,46]. DF fraction was made up of 81% of insoluble components and minor percentages of high molecular weight (HMW) and low molecular weight (LMW) soluble compounds (12.7% and 6.2% of TDF, respectively), in agreement with earlier studies [21]. In addition, it was observed that β-glucan was a representative polysaccharide of the soluble DF accounting for 33.05% of the total HMW-SDF (soluble dietary fiber) fraction. Starch was another abundant carbohydrate in WB that reached 19 g 100 g^−1^ values comparable to the reported by other authors [47]. Protein content was 15.6 g 100 g^−1^, a value that falls within the range found in the literature [9]. Ash content of WB was around 5 g 100 g^−1^, resulting in agreement with other studies [38,46]. The fat content was relatively low (4.0 g 100 g^−1^) in agreement with observations reported previously [9,11,44]. The WB oil fraction was composed mainly by polyunsaturated fatty acids (PUFA) (63.9% of total fatty acids), followed by monounsaturated fatty acids (MUFA) (19.7% of total fatty acids), and lower proportion of saturated fatty acids (SFA) (16.4% of total fatty acids). FA content in WB was 553.96 mg 100 g^−1^ being mainly present as the bound form (99%).

### 3.2. Effect of HT Time on Ferulic Acid (FA) and Total Phenolic (TP) Content, Total Antioxidant Capacity (TAC) and AIA of WB Hydrolysate

**Figure 2:**
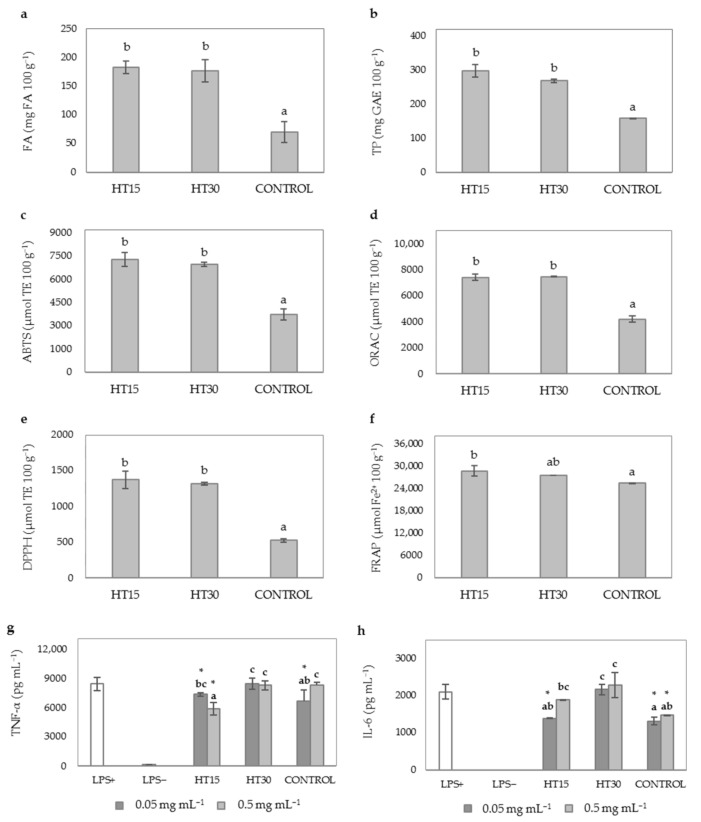
Evaluation of time during HT on phenolic compounds content, TAC and AIA of WB hydrolysates. (**a**) FA content (mg 100 g^−1^); (**b**) TP content (mg GAE 100 g^−1^); (**c**) ABTS scavenging activity (µmol TE 100 g^−1^); (**d**) ORAC (µmol TE 100 g^−1^); (**e**) DPPH scavenging activity (µmol TE 100 g^−1^); (**f**) FRAP (µmol Fe^2+^ 100 g^−1^); (**g**) TNF-α levels (pg mL^−1^); (**h**) IL-6 levels (pg mL^−1^). Sample identification: hydrolyzed WB without HT (CONTROL), hydrothermal treatment for 15 min (HT15), hydrothermal treatment for 30 min (HT30). Different lowercase letters show significant differences between different experimental groups (one-way ANOVA, post hoc Duncan’s test, *p* ≤ 0.05). * denote significant differences between mean values of cell treatments and untreated LPS+ cells (one-way ANOVA, post hoc Duncan’s test, *p* ≤ 0.05). Abbreviations: 2,2′-azino-bis (3-ethylbenzothiazoline-6-sulfonic acid) (ABTS); 2,2-diphenyl-1-picryl-hydrazyl-hydrate (DPPH); ferulic acid (FA); ferric reducing antioxidant power (FRAP); gallic acid equivalents (GAE); interleukin-6 (IL-6); lipopolysaccharide (LPS); oxygen radical absorbance capacity (ORAC); Trolox equivalents (TE); total phenolics (TP); tumor necrosis factor-alpha (TNF-α).

Different autoclave times (15 and 30 min) were evaluated for the HT and compared with no pretreatment assayed as control. HT experiments with WB (HT15 and HT30) affected positively to FA (Figure 2a) and TP content of WB hydrolysates that reached values two times higher than those found in control (non-thermally treated WB hydrolysate) (Figure 2b). The use of high temperature combined with pressure favored FA and TP release during hydrolysis, as previously reported by different authors [7,48]. HT may enhance solubilization of celluloses and hemicelluloses of WB increasing the content of extractable phenolic compounds due to the breakdown of the lignocellulosic fibers [49]. In particular, Deroover et al. [50] reported that autoclaving can increase more effectively the soluble extractable phenolic compounds, such as *p*-coumaric and FA, compared to other conventional methods such as boiling. No significant differences (*p* ≥ 0.05) were observed between HT15 and HT30; although, there was a reducing tendency in TP levels with longer autoclave times, which may be associated with the thermal instability of phenolic compounds [51].

To evaluate the TAC of WB hydrolysates, several analytical methods were used including DPPH, ORAC, ABTS, and FRAP assays. In general, HT significantly increased 2-fold the antioxidant activity of WB hydrolysates *(p* < 0.05) regardless of the method used. No significant differences were observed between the antioxidant activity of HT15 and HT30 (Figure 2c–f). This observation indicated that longer HT times (30 min) did not result in an improvement of the TAC of WB hydrolysates. This observation suggested that increasing HT time from 15 to 30 min did not caused a higher solubilization of DF components and, subsequently, did not allow a greater soluble FA (Figure 2a) and TP (Figure 2b) enzymatic release by Ultraflo XL.

HT significantly influenced the AIA of WB hydrolysates although a different behavior was observed depending on the duration of the HT (Figure 2g,h). Improvements in the AIA of WB hydrolysates were observed for HT15 at doses of 0.5 mg mL^−1^ (30.75% inhibition of TNF-α levels vs. 1.06% inhibition of control, Figure 2g). However, the ability of WB hydrolysates to reduce IL-6 levels (10.5 46% inhibition, Figure 2h) was not significantly improved when HT was applied as pretreatment for 15 min (HT15). Evidence from in vitro and in vivo studies have demonstrated the anti-inflammatory effects of FA and AX hydrolysis products including feruloyl oligosaccharides, arabinooligosaccharides and xylooligosaccharides [9,52]. In agreement with our study, previous investigations reported a noticeable increase of these anti-inflammatory compounds when thermal and enzymatic treatments are used in combination [9,23,53]. Therefore, the higher ability of HT15 to reduce TNF-α production in LPS induced macrophages could be attributed to the increased solubility of DF components caused by thermal-pressure treatments and the improved enzymatic release of WB bound phenolics and AX oligosaccharides by Ultraflo XL. In contrast, HT30 showed similar cytokine levels (Figure 2g,h) compared to untreated LPS+ cells. These results are indicative of the loss of the anti-inflammatory potential of WB hydrolysates when a shorter time (30 min) was applied. There are two plausible explanations of these results related to the thermal degradation of thermolabile phenolic compounds, firstly in consistency with the decreasing trend observed in TP content of HT30 as compared to HT15 (Figure 2b) and secondly the neogeneration of Maillard reaction products (MRP) in thermally treated products with processing time as it was previously observed in other studies [54]. MRPs lead to the formation of advanced-glycation (AGEs) and lipoxidation end products (ALEs) with proinflammatory activity. Moreover, MRPs could activate inflammatory responses via macrophages activation and immune dysfunction in helper T lymphocytes [55]. Therefore, all these studies could explain the loss of anti-inflammatory effects observed for HT30.

Given the higher yields of FA and TP, and antioxidant and anti-inflammatory potential of HT15, this operating time was selected for further experiments.

### 3.3. Effect of the Sequential Use of HT and HHP on FA and TP Content, TAC and AIA of WB Hydrolysate

**Figure 3:**
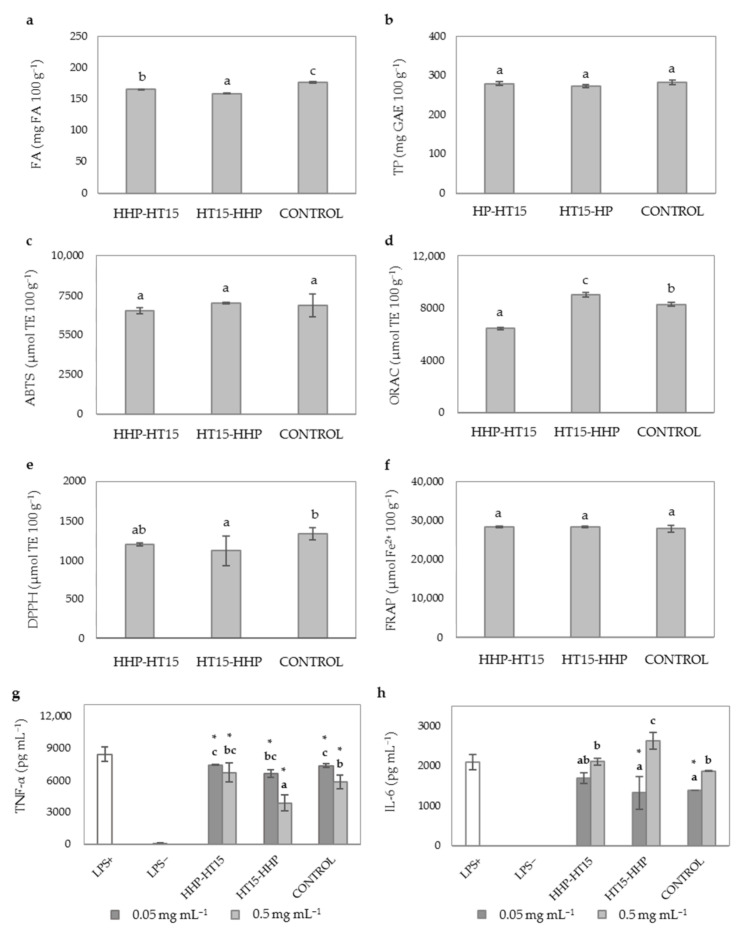
Evaluation of the sequential use of HT and HHP on phenolic compounds content, TAC and AIA of WB hydrolysates. (**a**) FA content (mg 100 g^−1^); (**b**) TP content (mg GAE 100 g^−1^); (**c**) ABTS scavenging activity (µmol TE 100 g^−1^); (**d**) ORAC (µmol TE 100 g^−1^); (**e**) DPPH scavenging activity (µmol TE 100 g^−1^); (**f**) FRAP (µmol Fe^2+^ 100 g^−1^); (**g**) TNF-α levels (pg mL^−1^); (**h**) IL-6 levels (pg mL^−1^). Sample identification: hydrothermal treatment for 15 min (CONTROL), HHP followed by HT for 15 min (HHP-HT15), and HT for 15 min followed by HHP (HT15-HHP). Different lowercase letters show significant differences between different treatment groups (one-way ANOVA, post hoc Duncan’s test, *p* ≤ 0.05). * denotes significant differences between mean values of cell treatments and untreated LPS+ cells (one-way ANOVA, post hoc Duncan’s test, *p* ≤ 0.05). Abbreviations: 2,2′-azino-bis (3-ethylbenzothiazoline-6-sulfonic acid) (ABTS); 2,2-diphenyl-1-picryl-hydrazyl-hydrate (DPPH); ferulic acid (FA); ferric reducing antioxidant power (FRAP); gallic acid equivalents (GAE); interleukin-6 (IL-6); lipopolysaccharide (LPS); oxygen radical absorbance capacity (ORAC); Trolox equivalents (TE); total phenolics (TP); tumor necrosis factor-alpha (TNF-α).

With the aim to study the efficiency of the sequential use of HT and HHP pretreatments in the enzymatic release of WB bound phenolics, HHP was applied before (HHP-HT15) and after (HT15-HHP) the HT and compared to HT15 treatment (CONTROL) (Figure 3). HHP has been used as the bound phenolic extraction method in a number of studies due to its increased mass transfer rate, cell membrane damage, and enhanced solvent permeability, improving production efficiency [8,56]. However, it has also been reported that HHP pretreatment at high pressure levels (6.08 × 10^8^ Pa) strengthened matrix network interactions thus hindering the release of bioactive compounds [57]. Our results on FA content of WB hydrolysates confirm this effect as FA yields were slightly reduced in HHP-HT15 and HT15-HHP as compared to CONTROL (Figure 3a). In addition, the pressurization at 6.08 × 10^8^ Pa for 5 min did not have any significant (*p* ≤ 0.05) effect on TP content of WB hydrolysates, neither after nor before the HT (Figure 3a,b). In agreement with our results, Pérez-Rodríguez et al. [53] demonstrated that the combination of HT and HHP led to lower FA levels in corn cob hydrolysates than those achieved with the single application of HT. This study also concluded that enzymatic FA release from corn cob is influenced by the interaction of temperature and time. Thus, a synergistic effect on the enzymatic liberation of FA by Ultraflo^®^ L was achieved when combination of HT treatment of corn cob was carried out at 130 °C/2 h followed by HHP at 6.08 × 10^8^ Pa/40 °C/15 min, operating conditions that differed greatly from the selected in the present study (autoclaving at 115 °C/15 min and pressurization at 6.08 × 10^8^ Pa/20 °C/5 min). 

As shown in Figure 3c–f, TAC of WB hydrolysates was not affected by the combination of HT and HHP pretreatments in consistency with results related to FA and TP content (Figure 3a,b respectively). Similarly, the AIA of WB hydrolysates was not modified after HHP-HT15 treatment as compared to CONTROL (Figure 3g,h), in line with results observed for FA and TP (Figure 3a,b). Interestingly, a shift in the order of application of both pretreatment methods (HT15-HHP) increased the ability of WB hydrolysate to reduce the levels of TNF-α at doses of 0.5 mg mL^−1^ (from 8400.0 to 3863.7 pg mL^−1^ that accounted for 54% inhibition, Figure 3g) as compared to CONTROL *(*from 8400.0 to 5817.9 pg mL^−1^ that corresponded to 30.74% inhibition) while no significant variations were observed for IL-6 (Figure 3h). The reason of this behavior is not completely clear since there is hardly any information about changes in lignin and hemicellulose during the sequential application of HHP and HT, and how these pretreatments affect to the enzymatic release of WB compounds with anti-inflammatory activity. When the first process unit is HT, a plausible mechanism for the improvement in the AIA of WB hydrolysates could be the swelling and thermal breakdown of the lignocellulosic materials, as previously described [58] that may promote, in a higher extent, the subsequent hydration and opening of the WB matrix during the HHP treatment, hence increasing the area for enzyme activity [59]. On the contrary, intense HHP as the first process unit could reduce swelling capacity of WB matrix [57] and hence, the polysaccharide solubilization efficiency of HT and the hydrolytic efficiency of enzymes might be reduced. This remains to be studied in further experiments.

In summary results from these experiments showed that application of HHP in the conditions used in the present study did not provide important improvements in the TP, TAC and AIA of WB hydrolysates. For this reason, further experiments were performed applying only HT15 as pretreatment for the subsequent enzymatic hydrolysis.

### 3.4. Effect of the Type of Organic Acid on FA and TP Contents, TAC and AIA of WB Hydrolysate

**Figure 4:**
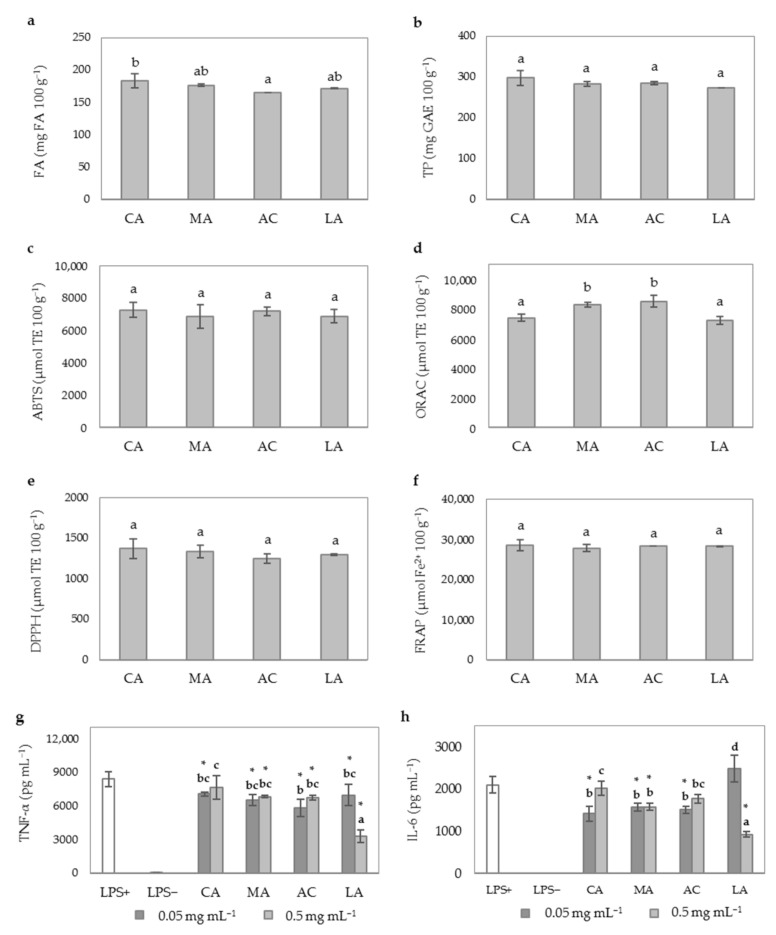
Effect of the type of organic acid used during WB hydrolysis by Ultraflo XL in optimal conditions (47 °C, pH 5, 20 h) on phenolic compounds content, TAC and AIA of WB hydrolysates. (**a**) FA content (mg 100 g^−1^); (**b**) TP content (mg GAE 100 g^−1^); (**c**) ABTS scavenging activity (µmol TE 100 g^−1^); (**d**) ORAC (µmol TE 100 g^−1^); (**e**) DPPH scavenging activity (µmol TE 100 g^−1^); (**f**) FRAP (µmol Fe^2+^ 100 g^−1^); (**g**) TNF-α concentration (pg mL^−1^); (**h**) IL-6 concentration (pg mL^−1^). In these experiments HT15 was used as a pretreatment. Sample identification: WB hydrolyzed in the presence of citric acid (CA), malic acid (MA), acetic acid (AC), and lactic acid (LA). Different lowercase letters show significant differences between samples (one-way ANOVA, post hoc Duncan’s test, *p* ≤ 0.05). * denotes significant differences between mean values of treatment groups and LPS+ cells (one-way ANOVA, post hoc Duncan’s test, *p* ≤ 0.05). Abbreviations: 2,2′-azino-bis (3-ethylbenzothiazoline-6-sulfonic acid) (ABTS); 2,2-diphenyl-1-picryl-hydrazyl-hydrate (DPPH); ferulic acid (FA); ferric reducing antioxidant power (FRAP); gallic acid equivalents (GAE); interleukin-6 (IL-6); lipopolysaccharide (LPS); oxygen radical absorbance capacity (ORAC); Trolox equivalents (TE); total phenolic content (TP); tumor necrosis factor-alpha (TNF α).

A recent study carried out in our laboratory demonstrated that when using Ultraflo XL, optimal pH should be maintained between 4.5 and 5.5 to maximize the FA yield, TAC and AIA of WB hydrolysates [9]. In the present study, it was compared the use of different organic acids as alternative to inorganic acids (HCl) during WB enzymatic treatment in keeping acidic conditions. Organic acids could be used as part of a green and safer processing strategy to produce a value-added functional ingredient. The type of organic acid used in the enzymatic treatment did not cause important changes in the enzymatic release of FA (Figure 4a) and TP (Figure 4b) from WB.

As shown in Figure 4c–f, similar results were observed for ABTS, ORAC, DPPH, and FRAP assays since non-significant differences were observed among WB treatments. Minor modifications were also noticed for the AIA of WB hydrolysates when different organic acids were used with the exception of LA (Figure 4g,h). WB hydrolysates at doses of 0.5 mg mL^−1^ containing LA showed the highest inhibition of TNF-α (from 8400.0 to 834.3 pg mL^−1^, accounting for 60.3% inhibition) and IL-6 levels (2102.1 to 926.8 pg mL^−1^ accounting for 55.9% inhibition). This finding is in line with results of an earlier study concluding that LA may inhibit LPS-activation of NFκβ, leading to downregulation of TNF-α and IL-6 in rat intestinal mucosa microvascular endothelial cells [60]. The recent study of Shan et al. [61] support previous evidence showing that LA is able to downregulate the expression of cytokines TNF-α and IL-12, secreted by M1-tumor associated macrophages. 

### 3.5. Effect of the Spray-Drying (SPD) Inlet Temperature on TP, TAC and AIA of WB Hydrolysate

In order to better address changes in the phenolic composition of hydrolyzed WB after SPD, a HPLC-ESI-QTOF/MS analysis was performed, and the list of phenolic compounds tentatively identified is summarized in Table 2, Figure S1. A total of thirteen phenolic compounds from three different phenolic classes including 10 phenolic acids, 2 flavonoids, and 1 hydroxybenzaldehyde were tentatively identified by comparison of retention time (RT), parent and fragment ion accurate masses, and molecular formulas with literature data. Compounds **1**, **2**, and **6** with theoretical [M − H]^−^ at *m/z* 154.1201 and RT of 4.3, 5.2, and 10.9 min were characterized as dihydroxybenzoic acid isomers (i1, i2, and i3, respectively) [62]. Compounds **3**, **4**, and **5** with theoretical [M − H]^−^ at *m/z* 137.0244 and RT of 7.1, 7.8, and 8.3 min were characterized as hydroxybenzoic acid isomers (i1, i2, and i3, respectively) [63]. A total of 4 hydroxycinnamic acids (Compounds **6**, **8**, **11,** and **13**) with detected [M − H]^−^ at *m/z* 353.0906, 179.0398, 193.0506, and 193.0505, were tentatively characterized as chlorogenic acid, caffeic acid, *trans-*FA and *cis-*FA, respectively. Previously, caffeic acid was identified in a thermally processed WB methanolic extract in the positive ionization mode [62] and *trans*-FA and *cis*-FA were identified in aleurone wheat fractions [63]. Regarding the flavonoid class, 2 flavones were found (Compounds **10** and **12**) tentatively characterized as isomers 1 and 2 of apigenin diglucosides with observed [M − H]^−^ at *m/z* 563.1447 and 563.1450, respectively [7,63]. At last, one hydroxybenzaldehyde (Compound **9**) with the molecular formula C_8_H_8_O_3_ and [M − H]^−^ at *m/z* 151.0389 was tentatively identified as vanillin, which was previously identified in purple wheat by HPLC-DAD [64].

Regardless of the temperature used in the SPD method, *trans*-FA was the major phenolic compound (202.04–245.84 mg 100 g^−1^) accounting for 97% of the total quantified phenolic compounds in WB hydrolysates (Table 3). Smaller amounts of *cis-*FA (1.71–1.94 mg 100 g^−1^) and isomers of apigenin diglucosides (1.30–1.52 mg 100 g^−1^ for isomer 1, and 1.60–1.80 mg 100 g^−1^ for isomer 2) were also accounted (Table 3). No significant effect of the inlet temperature on any of the quantified phenolic compounds was observed among treatments except for *trans*-FA values that showed a significant decrease at 160 °C. These results suggest that phenolic compounds present in WB hydrolysates were stable at 130–140 °C inlet temperature. Consistent with our results, Cheng et al. [65] reported that five phenolic compounds, among them FA, remained stable at temperatures up to 150 °C for at least 20 min. However, at 200 °C FA and protocatechuic acid were almost completely degraded. SPD was reported to be a suitable technique for drying heat sensitive polyphenols, although operating temperatures seemed to be very important. Drying inlet/outlet temperatures (150/80 °C, respectively) were associated with high retention rates of TP and anthocyanins in blackberry juice [28]. On the other hand, different combinations of inlet/outlet temperatures in rice bran extract powder showed that increases in the inlet/outlet temperatures (100/85 to 120/89 °C and 140/92 °C) negatively affected the concentration of TP [66].

**Figure 5:**
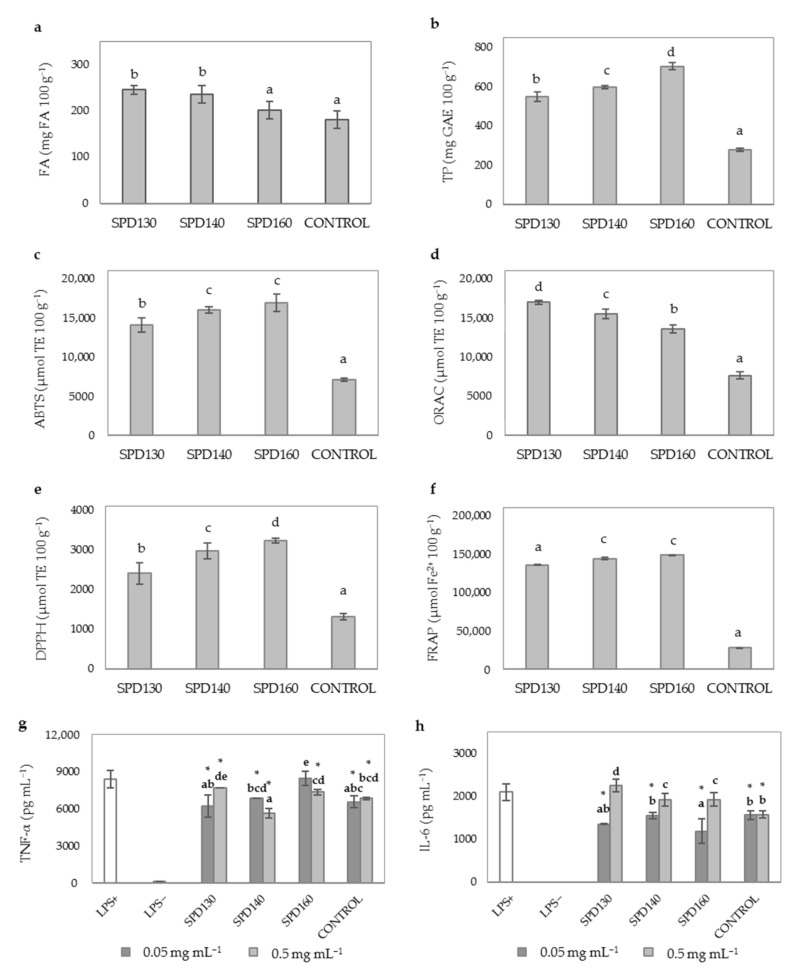
Evaluation of the SPD inlet temperature on phenolic compounds content, TAC and AIA of WB hydrolysates. (**a**) FA content (mg 100 g^−1^); (**b**) TP content (mg GAE 100 g^−1^); (**c**) ABTS scavenging activity (µmol TE 100 g^−1^); (**d**) ORAC (µmol TE 100 g^-1^); (**e**) DPPH scavenging activity (µmol TE 100 g^-1^); (**f**) FRAP (µmol Fe^2+^ 100 g^-1^); (**g**) TNF-α levels (pg mL^−1^); (**h**) IL-6 levels (pg mL^−1^). Sample identification: hydrolyzed WB treated at HT15 (CONTROL), hydrolyzed WB spray-dried at 130 °C (SPD130), 140 °C (SPD140), or 160 °C (SPD160). Different lowercase letters show significant differences between samples (one-way ANOVA, post hoc Duncan’s test, *p* ≤ 0.05). * indicates significant differences for mean values relative to treated cells (LPS+) (one-way ANOVA, post hoc Duncan’s test, *p* ≤ 0.05). Abbreviations: Abbreviations: 2,2′-azino-bis (3-ethylbenzothiazoline-6-sulfonic acid) (ABTS); 2,2-diphenyl-1-picryl-hydrazyl-hydrate (DPPH); ferulic acid (FA); ferric reducing antioxidant power (FRAP); gallic acid equivalents (GAE); interleukin-6 (IL-6); lipopolysaccharide (LPS); oxygen radical absorbance capacity (ORAC); Trolox equivalents (TE); total phenolics (TP); tumor necrosis factor-alpha (TNF-α).

TP content significantly (*p* ≤ 0.05) increased after SPD as compared to CONTROL (Figure 5b). There was an increasing tendency in the TP content of WB hydrolysates with increasing inlet temperatures, thus SPD160 showed the highest values. These findings were not coincident with the phenolic quantitative analysis carried out by mass spectrometry indicating that the increased TP content observed after SPD may be due to the formation of melanoidins and other MRPs that have reducing power and may interfere in the analysis of TP as determined by the Folin–Ciocalteu’s reagent [67].

The effect of the use of different inlet temperatures during SPD on the TAC of WB hydrolysates was also studied and compared with control (Figure 5c–f). An increasing trend was observed for the TAC in WB hydrolysates after SPD at different temperatures, with the exception of ORAC values that showed an inverse behavior (Figure 5d). The increasing trend in this capacity with increasing temperature can be associated with a higher generation of MRPs, since HPLC-ESI-QTOF/MS analyses did not show differences in the amounts of TP at 130–140 °C inlet temperature (Table 3). The molecular weight of the melanoidins produced after Maillard reactions depends on temperature applied [68,69,70]. SPD treatments at higher temperatures (160 °C) may have favored the formation of HMW melanoidins with higher antioxidant capacity, as previously reported [68,69,70]. An exception to the observations described above was the ORAC assay that showed an inverse behavior that could be associated to the peroxyl radical inhibitory activity of HMW melanoidins increasing with higher temperature and processing time (Figure 5d). 

Differences in the AIA of WB hydrolysates after SPD at different inlet temperature were hardly observed (Figure 5g,h). In general, SPD did not affect the ability of WB hydrolysates to reduce TNF-α irrespective of the inlet temperature tested. A significant improvement in the inhibition of the latter cytokine was only observed when cells were treated with SPD140 at 0.5 mg mL^−1^ (60.3% inhibition). In contrast, SPD negatively affected the inhibitory action of WB hydrolysates on IL-6 levels when macrophages were exposed at doses of 0.5 mg mL^−1^. SPD160 was an exception to this observation as cell treatments at 0.05 mg mL^−1^ of this WB hydrolysates showed a higher reduction in the production of this cytokine. 

Water activity (a_w_) of dried WB hydrolysates in all tested conditions was under 0.32 and SPD130 showed the lowest values (Table 4) although the differences were not significant between treatments. This could be explained by the loss of water binding structures with increasing temperatures, while water content remaining similar, resulting in an inversely proportional relation between aw and inlet air temperature. Water molecules trapped inside macromolecules or immobilized by hydrogen binding require more energy to be evaporated [71]. 

Total solids yield after SPD of WB soluble fraction at different inlet temperature is shown in Table 5. This parameter increased with increasing inlet temperatures up to 140 °C, due to the improvement of droplet drying and reduced droplet/particle deposition on the walls of the drying chamber. Higher inlet temperatures (160 °C) decreased sharply total solids content as reported previously [72]. However, although the use of lower temperature in the inlet SPD showed lower yields, those values were duplicated when the HHP was included in the process (Table 5). For this reason, since a lower temperature can contribute with the reduction of energy, an important issue in the dry processes can reduce the potential formation of Maillard derivatives. The results showed that the use of 130 °C inlet temperature will be more successful for the stabilization of WB hydrolysates.

### 3.6. Effect of MEC on the FA and TP Content, TAC and AIA of WB Hydrolysate

**Figure 6:**
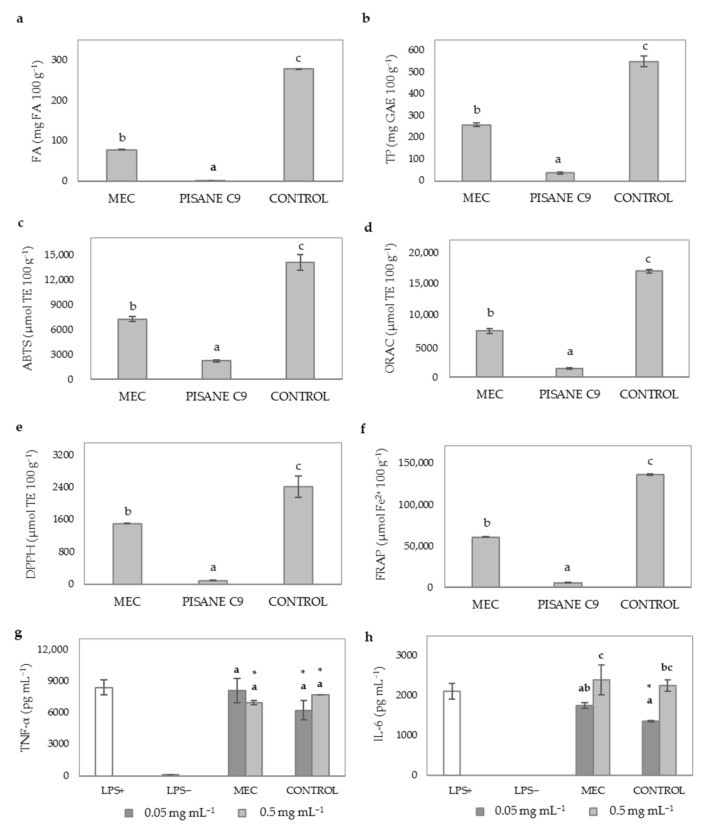
Evaluation of MEC on phenolic compounds content, TAC and AIA of WB hydrolysates. (**a**) FA content (mg 100 g^−1^); (**b**) TP content (mg GAE 100 g^−1^); (**c**) ABTS scavenging activity (µmol TE 100 g^−1^); (**d**) ORAC (µmol TE 100 g^−1^); (**e**) DPPH scavenging activity (µmol TE 100 g^−1^); (**f**) FRAP (µmol Fe^2+^ 100 g^−1^); (**g**) TNF-α levels (pg mL^−1^); (**h**) IL-6 levels (pg mL^−1^). Sample identification: hydrolyzed WB spray-dried at 130 °C (CONTROL); pea protein (Pisane C_9_); hydrolyzed WB microencapsulated with Pisane C_9_ and spray dried at 130 °C (MEC). Different lowercase letters show significant differences between samples (one-way ANOVA, post hoc Duncan’s test, *p* ≤ 0.05). * denotes significant differences between mean values of treatment groups and LPS+ cells (one-way ANOVA, post hoc Duncan’s test, *p* ≤ 0.05). Abbreviations: 2,2′-azino-bis (3-ethylbenzothiazoline-6-sulfonic acid) (ABTS); 2,2-diphenyl-1-picryl-hydrazyl-hydrate (DPPH); ferulic acid (FA); ferric reducing antioxidant power (FRAP); gallic acid equivalents (GAE); interleukin-6 (IL-6); lipopolysaccharide (LPS); oxygen radical absorbance capacity (ORAC); Trolox equivalents (TE); total phenolic content (TP); tumor necrosis factor-alpha (TNF-α).

With the aim of increasing stability of the phenolic compounds in WB hydrolysates, MEC was performed using pea protein (Pisane C_9_) and SPD at 130 °C. The results showed that MEC had 50% less FA and TP content than control (SPD130) (Figure 6a,b). The MEC process may have little detrimental effect of the bioactive compound abundancy, since this observed difference well reflects the ratio used for the coprecipitation (1:1; SPD130 extract:Pisane C_9_) (Figure 6c–g).

Finally, the effect of MEC on AIA of WB hydrolysates was studied (Figure 6g,h). Doses of 0.5 mg mL^−1^ of MEC reduced TNF-α concentration in a similar way compared to CONTROL. Taking this into account, that MEC contains SPD130 in a proportion of 1:1, our results suggest that the AIA is likely related to the concentration of WB hydrolysate presented in cell treatments.

### 3.7. Relative Antioxidant Capacity Index (RACI) and Principal Component Analysis (PCA) 

RACI was calculated to obtain a complete and dynamic measurement of the TAC, a theoretical concept calculated from the perspective of statistics by integrating the antioxidant capacity values generated from different in vitro methods (Table S1).

Correlation analysis (Figure S2) showed a significant relationship between TP and antioxidant capacity (for ABTS: *r* = 0.99, *p* < 0.05; for ORAC: *r* = 0.95, *p* < 0.05; DPPH: *r* = 0.99, *p* < 0.05; and FRAP: *r* = 0.99, *p* < 0.05). The high correlation between antioxidant capacity and TP content after the HT may reflect the fact that covalently bound phenolic acids such as FA are the major components released in the obtained final ingredient.

**Figure 7:**
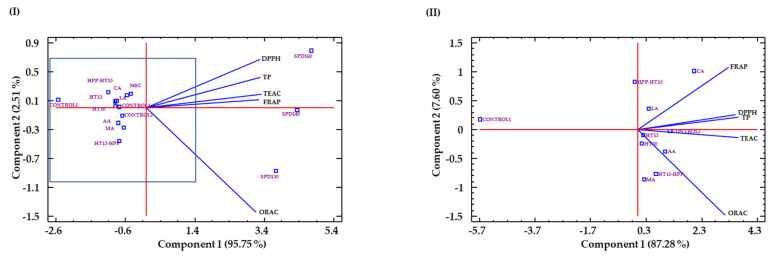
Biplots of (**I**) PCA analysis of antioxidant results of hydrolyzed WB and (**II**) PCA analysis of the samples within the inner rectangle, i.e., excluding SPD samples. Sample identification: hydrolyzed WB without hydrothermal treatment (CONTROL), with hydrothermal treatment for 15 min (HT15), with hydrothermal treatment for 30 min (HT30), with hydrothermal treatment for 15 min and HHP (HT15-HHP), with HHP and hydrothermal treatment for 15 min (HHP-HT15), treated with citric acid (CA), malic acid (MA), acetic acid (AC), lactic acid (LA), spray-dried at 130 °C (SPD130), 140 °C (SPD140), 160 °C (SPD160), and microencapsulated with Pisane C_9_ at 130 °C (MEC). Abbreviations: 2,2′-azino-bis (3-ethylbenzothiazoline-6-sulfonic acid) (ABTS); 2,2-diphenyl-1-picryl-hydrazyl-hydrate (DPPH); ferric reducing antioxidant power (FRAP); oxygen radical absorbance capacity (ORAC); total phenolics (TP).

The use of PCA (Figure 7I) showed that the first component (PC1) explained 95.75% of the variance and 2.51% of the second (PC2), being the total explained variance of 97.28%. The variables antioxidant capacity (ABTS, DPPH, and FRAP) and TP content were well correlated with PC1, and antioxidant capacity against ORAC was better correlated to PC2. PC1 allowed the differentiation of SPD ingredients from MEC ingredients or not dried; meanwhile, PC2 allowed to separate SPD at 130 °C from SPD at 140 °C and 160 °C, being this one highly correlated with ORAC values.

A second PCA analysis was carried out (Figure 7II) with the samples in the inner rectangle shown in Figure 7I, i.e., excluding SPD samples, which were clearly discriminated in the first PCA. The first component (PC1) allowed one to differentiate the control sample from the rest of the treatments, which showed higher antioxidant results in all parameters. The second component (PC2) allowed clear differentiation of samples treated with HHP before HT (HHP-HT15) from those treated with HHP after HT (HT15-HHP), mostly based on differences in FRAP and ORAC values. In accordance to the PCA results, SPD130 and HT15-HHP ingredients would have higher affinity for the peroxyl radicals (ORAC value) and, therefore, better ability for breaking fatty acid oxidative chain reactions, as compared to the rest of the ingredients obtained.

### 3.8. Selection of Processing Conditions and Chemical Characterization of Final Products 

Following the definition of de Figueirêdo et al. [73] who consider that a well-designed extractive process combines the lowest specific energy consumption and the least loss of solvent, HT for 15 min, HHP, and SPD at 130 °C would reduce both the temperature of thermal treatment and spray-drying without reduction of bioactive properties, and reaching a yield close to 50%. The use of MEC did not produce a decrease on the bioactive properties and since this technology may increase its stability during storage and digestion, the incorporation of this process can be positive. Moreover, pea protein can even add additional advantages during the digestion since the hydrolysis of pea protein could potentially provide additional bioactive properties from the peptides released during gastrointestinal digestion. On the other hand, there are some limitations with this technique, such as the limited availability of coating materials that can be used for applications, and the thermolability of core materials during the SPD process. For all these reasons, SP130 and MEC were selected as the most suitable processing conditions and their chemical composition of resulting end products is compared in Table 6. Nutritional composition of Pisane C_9_ is also included to explain differences between the two final products.

TDF was the main component in SPD130 (15.21 ± 0.58 g 100 g^−1^) that was composed exclusively of soluble poly-(HMW-SDF) and oligosaccharides (LMW-SDF). IDF was not detected because of the enzymatic solubilization of WB celluloses and hemicelluloses catalyzed by Ultraflo XL [9] and the subsequent filtration of WB hydrolysates for the removal of the insoluble fraction (Figure 1). As compared to MEC, HMW-SDF represented more than 50% of TDF in SPD130 (64.04%) and MEC (84.00%). However, the LMW-SDF and β-glucan content in SPD130 were double as compared to MEC. Starch values were significantly higher in SPD130 than in MEC. On the other hand, addition of Pisane C_9_ as a wall material in MEC had a significant effect on its protein and phytic acid contents. Protein was the major compound of this powder, being 3.7-times higher compared to SPD130 values due to the addition of Pisane C_9_ (81.70% protein). A higher phytic acid concentration was observed for MEC compared to SPD130, this rise was probably due to the utilization of Pisane C_9_ (4.05 g of phytic acid 100 g^−1^) as wall material. Finally, MEC exhibited lower values of soluble phenolic compounds and FA isomers than SPD130.

## 4. Conclusions

In the present study, WB processing was developed based in a multistep process at a 2 L scale comprising pressure and thermal pretreatments followed by enzymatic hydrolysis with Ultraflo XL and SPD and MEC were used to stabilized the bioactive compound. The solubilization of FA and TP compounds, TAC and AIA were monitored at all steps. Results showed that the use of HT enhanced the enzymatic-assisted extraction of bound phenolic compounds in WB although 15 min of treatment seemed to be enough to obtain the highest phenolic yields, TAC, and AIA, which was desirable to reduce the energetic cost of the process. On the other hand, although the use of HHP did not produce an enhancement in the evaluated bioactive properties, HHP had a positive effect on the total solids yield. WB enzymatic treatment with Ultraflo XL in the presence of LA improved the anti-inflammatory efficiency of hydrolysates. SPD inlet temperature did not affect phenolic composition although it increased antioxidant activity and anti-inflammatory activity in specific operating conditions. MEC with Pisane C_9_ reduced the absolute amounts of phenolics and TAC and did not provide additional improvements in the anti-inflammatory activity of the final product. Overall, the most energy efficient and scalable processing route for maximum FA yield and bioactive properties was HT for 15 min-enzymatic hydrolysis with LA-SPD at 130 °C. In addition to the final bioactive and soluble WB powder, the enzymatic treatment generated a solid residue probably containing IDF and proteins that could be further exploited for more valuable applications, such as the production of ingredients for food (given that the proposed processes do not involve the use of solvents or toxic reagents).

## Figures and Tables

**Table 1:** Chemical composition of wheat (*Triticum aestivum* L. var. Craklin) bran.

WB Components	Mean ± Standard Deviation
TDF (g 100 g^−1^ d.m.)	46.64 ± 0.01
IDF (g 100 g^−1^ d.m.)	37.83 ± 0.40
HMW-SDF (g 100 g^−1^ d.m.)	5.90 ± 0.40
LMW-SDF (g 100 g^−1^ d.m.)	2.91 ± 0.01
β-glucan (g 100 g^−1^ d.m.)	1.95 ± 0.10
Starch (g 100 g^−1^ d.m.)	19.12 ± 0.56
Protein (g 100 g^−1^ d.m.)	15.62 ± 0.09
Ash (g 100 g^−1^ d.m.)	4.98 ± 0.05
Fat (g 100 g^−1^ d.m.)	4.04 ± 0.46
SFA (% of total fatty acids)	16.38 ± 0.01
MUFA (% of total fatty acids)	19.67 ± 0.30
PUFA (% of total fatty acids)	63.95 ± 0.32
Phytic acid (g 100 g^−1^ d.m.)	2.82 ± 0.07
Total phenolic compounds	1.23 ± 0.09
Free (g 100 g^−1^ d.m.)	0.26 ± 0.01
Bound (g 100 g^−1^ d.m.)	0.97 ± 0.07
Total FA (mg 100 g^−1^ d.m.)	553.96 ± 60.13
Free (mg 100 g^−1^ d.m.)	5.9 ± 1.09
Bound (mg 100 g^−1^ d.m.)	547.93 ± 50.05

Data are mean values ± standard deviation of two replicates. Abbreviations: total dietary fiber (TDF); insoluble dietary fiber (IDF); high molecular weight soluble dietary fiber (HMW-SDF); low molecular weight soluble dietary fiber (LMW-SDF); monounsaturated fatty acids (MUFA); polyunsaturated fatty acids (PUFA); saturated fatty acids (SFA); ferulic acid (FA).

**Table 2:** Chromatographic and mass spectrum data of the phenolic compounds identified in spray-dried (SPD) WB hydrolysates.

No.	Tentative Identification	Molecular Formula	RT (min)	Observed (*m/z*)	Score	Mass Error (ppm)
	**Phenolic acids**					
	** Hydroxybenzoic acids**					
1	2,4-dihydroxybenzoic acid (i1)	C_7_H_6_O_4_	4.30	153.0192	76.7	−0.76
2	2,4-dihydroxybenzoic acid (i2)	C_7_H_6_O_4_	5.20	153.0197	84.67	−2.78
3	4-hydroxybenzoic acid (i1)	C_7_H_6_O_3_	7.10	137.0251	87.33	−6.2
4	4-hydroxybenzoic acid (i2)	C_7_H_6_O_3_	7.80	137.0279	96.87	−2.75
5	4-hydroxybenzoic acid (i3)	C_7_H_6_O_3_	8.30	137.0242	90.06	0.15
7	2,4-dihydroxybenzoic acid (i3)	C_7_H_6_O_4_	10.9	153.0194	99.74	−3.46
	** Hydorxycinnamic acids**					
6	Chlorogenic acid	C_16_H_18_O_9_	10.30	353.0906	64.68	−4.95
8	Caffeic acid	C_9_H_8_O_4_	11.40	179.0398	65.41	−13.96
11	*Trans* ferulic acid	C_10_H_10_O_4_	20.10	193.0506	99.75	−0.07
13	*Cis* ferulic acid	C_10_H_10_O_4_	21.8	193.0505	98	0.93
	**Flavonoids**					
	** Flavones**					
10	Apigenin diglucoside (i1)	C_26_H_28_O_14_	19.00	563.1447	63.87	−8.53
12	Apigenin diglucoside (i2)	C_26_H_28_O_14_	20.70	563.1450	68.00	−8.36
	**Other polyphenols**					
	** Hydroxybenzaldehydes**					
9	Vanillin	C_8_H_8_O_3_	15.20	151.0408	78.24	−3.46

Abbreviations: retention time (RT); isomer (i).

**Table 3:** Quantification (mg 100 g^−1^ d.w.) of tentatively identified phenolic compounds by HPLC-ESI-QTOF/MS of WB hydrolysates spray-dried at different inlet temperatures.

Phenolic Compounds	SPD130	SPD140	SPD160	Standard for Quantification
Apigenin diglucoside isomers	2.90 ± 0.04 ^a^	3.23 ± 0.01 ^a^	3.28 ± 0.06 ^a^	Vitexin ^1^
Caffeic acid	tr	tr	tr	Caffeic acid ^2^
Chlorogenic acid	tr	tr	tr	Chlorogenic acid ^3^
*Cis-*FA	1.86 ± 0.60 ^a^	1.71 ± 0.31 ^a^	1.94 ± 0.16 ^a^	Ferulic acid ^4^
2,4-dihydroxybenzoic acid isomers	tr	tr	tr	2,4-dihydroxybenzoic acid ^5^
4-hydroxybenzoic acid isomers	tr	tr	tr	4-hydroxybenzoic acid ^6^
*Trans*-FA	245.84 ± 9.83 ^b^	236.11 ± 18.64 ^b^	202.04 ± 18.60 ^a^	Ferulic acid ^4^
Vanillin	tr	tr	tr	Vanillin ^7^

Data are mean values ± standard deviation of two replicates. Different letters in the same row indicate significant differences among mean values of different treatments (one-way ANOVA, post hoc Duncan’s test, *p* ≤ 0.05). Abbreviations: ferulic acid (FA); traces (tr); hydrolyzed WB spray-dried at 130 °C (SPD130), at 140 °C (SPD140), and at 160 °C (SPD160). ^1^ Linearity range 0–25 µg mL^−1^, *R*^2^ = 1.00, LOD = 0.22 μg mL^−1^, LOQ = 0.67 μg mL^−1^. ^2^ Linearity range 0–2.77 µg mL^−1^, *R*^2^ > 0.99, LOD = 0.16 μg mL^−1^, LOQ = 0.47 μg mL^−1^. ^3, 7^ Linearity range 0–3.75 µg mL^−1^, *R*^2^ = 1.00. ^4^ Linearity range 0–25 µg mL^−1^, *R*^2^ = 1.00, LOD = 0.04 μg mL^−1^, LOQ = 0.12 μg mL^−1^. ^5^ Linearity range 0.23–3.75 µg mL^−1^, *R*^2^ = 1.00. ^6^ Linearity range 0–3.75 µg mL^−1^, *R*^2^ > 0.99.

**Table 4:** Water activity (a_w_) of hydrolyzed WB powders obtained by SPD at different inlet temperatures.

Hydrolyzed WB	a_w_
SPD130	0.21 ± 0.10 ^a^
SPD140	0.31 ± 0.03 ^a^
SPD160	0.27 ± 0.08 ^a^

Data are mean values ± standard deviation of two replicates. Different lowercase letters show significant differences between samples (one-way ANOVA, post hoc Duncan’s test, *p* ≤ 0.05). Abbreviations: hydrolyzed WB spray-dried at 130 °C (SPD130), 140 °C (SPD140), or 160 °C (SPD160).

**Table 5:** Total solids yield (g 100 g^−1^) after SPD of WB hydrolysates at different inlet temperature.

Hydrolyzed WB	Yield (g 100 g^−1^ d.m.)
SPD130 (with HHP)	53
SPD130 (without HHP)	22
SPD140 (without HHP)	36
SPD160 (without HHP)	26

Data are mean values ± standard deviation of two replicates. Abbreviations: hydrolyzed WB spray-dried at 130 °C (SPD130), 140 °C (SPD140), or 160 °C (SPD160), HHP (high hydrostatic pressure).

**Table 6:** Proximate composition of powdered WB hydrolysates obtained using SPD at 130 °C.

Proximate Composition	SPD130	MEC	Pisane C_9_
TDF (g 100 g^−1^ d.m.)	15.21 ± 0.58 ^a^	14.32 ± 0.39 ^a^	1.4 *
IDF (g 100 g^−1^ d.m.)	ND	ND	-
HMW-SDF (g 100 g^−1^ d.m.)	9.74 ± 1.34 ^a^	12.03 ± 0.97 ^a^	1.4 *
LMW-SDF (g 100 g^−1^ d.m.)	5.48 ± 0.73 ^b^	2.28 ± 0.58 ^a^	-
β-glucan (g 100 g^−1^ d.m.)	2.50 ± 0.23 ^b^	1.35 ± 0.15 ^a^	-
Starch (g 100 g^−1^ d.m.)	10.28 ± 0.72 ^b^	1.39 ± 0.04 ^a^	
Protein (g 100 g^−1^ d.m.)	10.36 ± 0.18 ^a^	39.18 ± 0.87 ^b^	81.7 *
Phytic acid (g 100 g^−1^ d.m.)	0.85 ± 0.10 ^a^	1.49 ± 0.08 ^b^	4.05 ± 0.07
Soluble phenolic compounds (g 100 g^−1^ d.m.)	0.93 ± 0.03 ^b^	0.60 ± 0.01 ^a^	0.20 ± 0.01
Soluble FA isomers (mg 100 g^−1^ d.m.)	248.94 ± 9.85 ^b^	102.70 ± 2.79 ^a^	ND

Data are mean values ± standard deviation of two replicates. Different lowercase letters show significant differences between samples (one-way ANOVA, post hoc Duncan’s test, *p* ≤ 0.05). * data according to the manufacturer’s product sheet. Abbreviations: ferulic acid (FA); high molecular weight soluble dietary fiber (HMW-SDF); low molecular weight soluble dietary fiber (LMW-SDF); not detected (ND); hydrolyzed WB spray-dried at 130 °C (SPD130); hydrolyzed WB microencapsulated with Pisane C_9_ and spray-dried at 130 °C (MEC).

## Data Availability

Data is contained within the article and supplementary material.

## Supplementary Materials

The following are available online at https://www.mdpi.com/article/10.3390/antiox10060969/s1, Table S1: Relative antioxidant capacity (RACI) for hydrolyzed WB; Figure S1: Pearson correlation analysis for hydrolyzed WB; Figure S2: Effect of different hydrolyzed WB on cell viability; Figure S3: Based peak chromatogram of the phenolic compounds identified in spray-dried (SPD) WB hydrolysates by HPLC-ESI-QTOF-MS (numbers are the same reported in Table 2).
