# Supplementary material for: A Novel Strategy to Produce a Soluble and Bioactive Wheat Bran Ingredient Rich in Ferulic Acid
Source: Antioxidants (Basel). 2021 Jun 16;10(6):969. doi: 10.3390/antiox10060969 (PMC8234745; doi:10.3390/antiox10060969)
Supplement: Supplementary file 1 [file antioxidants-10-00969-s001.zip › antioxidants-1250063-supplementary.pdf]

Table S1. Relative antioxidant capacity (RACI) for hydrolysed WB.

Hydrolysed WB	RACI
CONTROL	-1,15
HT15	-0,471
HT30	-0,470
CA	-0,332
MA	-0,432
AA	-0,395
LA	-0,480
HHP-HT15	-0,558
HT15-HHP	-0,427
SPD130	1,449
SPD140	1,701
SPD160	1,860
MEC	-0,291

Sample identification: without hydrothermal treatment (CONTROL), with hydrothermal treatment for 15 min (HT15), with hydrothermal treatment for 30 min (HT30), with hydrothermal treatment for 15 min and HHP (HT15-HHP), with HHP and hydrothermal treatment for 15 min (HHP-HT15), treated with citric acid (CA), malic acid (MA), acetic acid (AC), lactic acid (LA), spray dried at 130 °C (SPD130), 140 °C (SPD140), 160 °C (SPD140), and microencapsulated with Pisane C_9 at 130 °C (MEC).

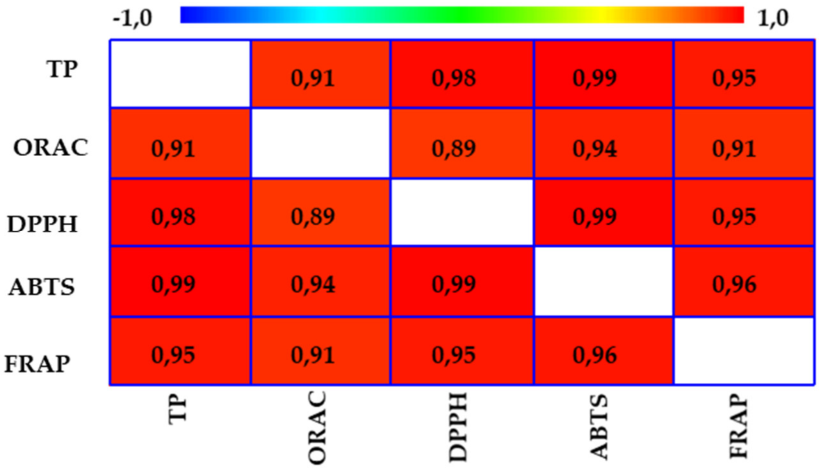

Figure S1. Pearson's correlation analysis for hydrolysed WB. Abbreviations: 2,2′-azino-bis (3-ethylbenzothiazoline-6-sulfonic acid) (ABTS); 2,2-diphenyl-1-picryl-hydrazyl-hydrate (DPPH); ferric reducing antioxidant power (FRAP); oxygen radical absorbance capacity (ORAC); total phenolic content (TP).

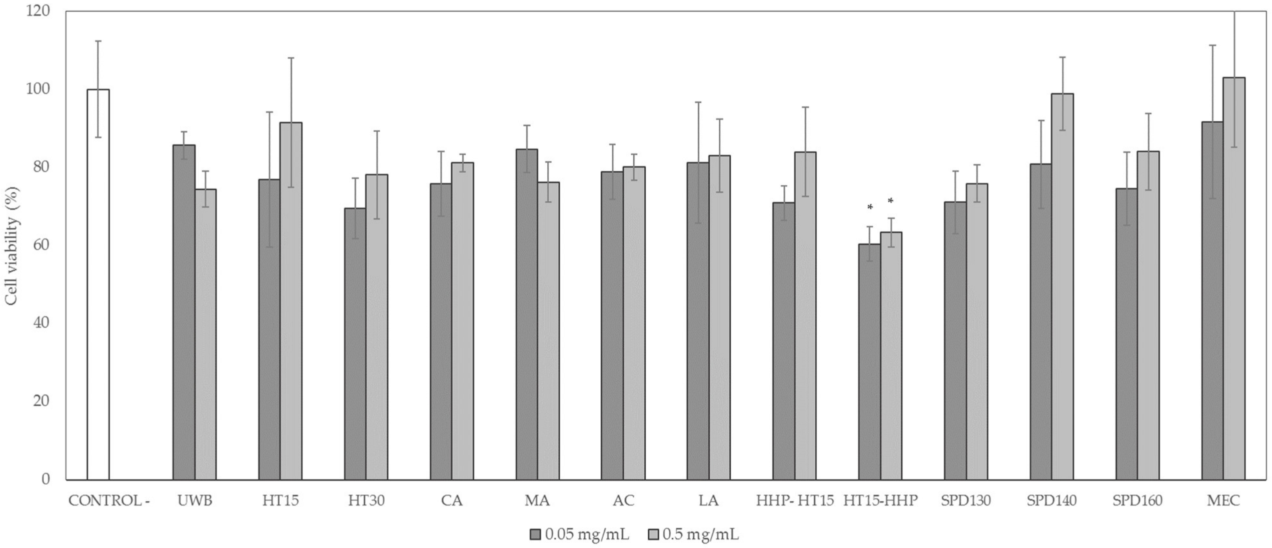

Figure S2. Effect of different hydrolysed WB on cell viability. Data are mean values (expressed as percentage relative to untreated cells) ± standard deviation of five replicates. * indicates significant differences for mean values relative to control cells (0 mg mL-1) (one-way ANOVA, post hoc Duncan´s test, $p \leq 0.05$). Abbreviations: acetic acid (AC); citric acid (CA); control (CT); high hydrostatic pressure (HP); hydrothermal treatment for 15 minutes (HT15); hydrothermal treatment for 30 minutes (HT30); lactic acid (LA); malic acid (MA); microencapsulation with Pisane C_9 at 130°C (MEC); spray drying at 130°C (SPD130); spray drying at 140°C (SPD140); spray drying at 160°C (SPD160); untreated wheat bran (UT-WB).

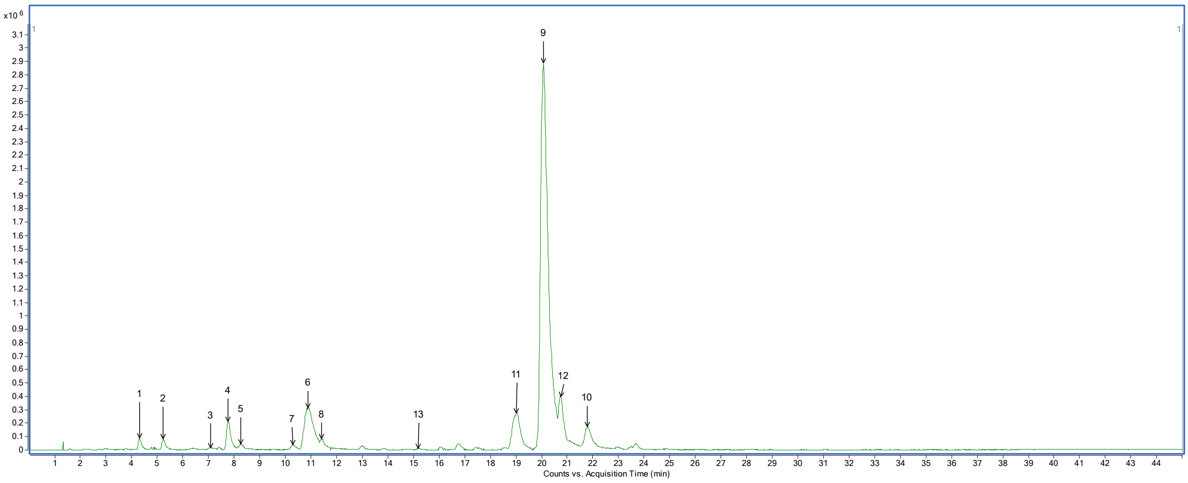

Figure S3. Based peak chromatogram of the phenolic compounds identified in spray dried (SPD) WB hydrolysates by HPLC-ESI-QTOF-MS (numbers are the same assigned to the identified compounds collected in Table 2).
